# Poly (ADP-Ribose) Polymerase 1 Mediated Arginase II Activation Is Responsible for Oxidized LDL-Induced Endothelial Dysfunction

**DOI:** 10.3389/fphar.2018.00882

**Published:** 2018-08-15

**Authors:** Qi Wang, Tong Zhao, Wei Zhang, Wenbin Yu, Bin Liu, Zhaoyang Wang, Wen Qiao, Qinghua Lu, Aihua Wang, Mingxiang Zhang

**Affiliations:** ^1^The Key Laboratory of Cardiovascular Remodeling and Function Research, Chinese Ministry of Education and Chinese Ministry of Public Health, Qilu Hospital of Shandong University, Jinan, China; ^2^Department of Cardiology, The Second Hospital of Shandong University, Jinan, China; ^3^Department of General Surgery, Qilu Hospital of Shandong University, Shandong, China; ^4^Department of Neurology, Qianfoshan Hospital of Shandong University, Shandong, China; ^5^Department of Pharmacology, College of Pharmacy, Xinxiang Medical University, Xinxiang, China

**Keywords:** PARP1, arginase II, oxLDL, endothelial function, atherosclerosis

## Abstract

It is well known that arginase II leads to decreased synthesis of nitric oxide (NO) by competing with endothelial nitric oxide synthase (eNOS) for their same substrate L-arginine. However, the regulatory mechanisms of arginase II production remain unclear. In this study, we hypothesized that poly- (ADP-ribose) transferase/polymerase-1 (PARP-1) may be a critical factor responsible for ox-LDL (oxidized Low Density Lipoprotein)-enhanced arginase II activity. We used serial deletions within plasmid constructs and found that a core promoter region of arginase II was located at the element of -774 to -738 bp and PARP-1 was identified specifically binding to this region. Inhibition of PARP-1 markedly reduced the endogenous arginase II expression and enhanced eNOS and NO production. Similarly, ox-LDL-induced increase in arginase II production and eNOS and NO reduction was substantially abolished by PARP-1 inhibition both *in vitro* and *in vivo*. Significant decrease in arginase II expression and increase in eNOS expression and NO levels, as well as improved endothelial function were observed in PARP-1-/- mice. The underlying mechanisms of ox-LDL-induced changes of PARP-1 expression involved migration of phosphorylated ERK2 into nuclei and direct interaction with PARP-1 which dramatically enhanced PARP-1 production, followed by histone acetylation to activate arginase II transcription process. Our studies demonstrated for the first time that PARP-1 regulates basal transcription process and ox-LDL-induced up-regulation of arginase II. These results demonstrated that PARP-1 offers a promising therapeutic target for endothelial dysfunction and atherosclerosis.

## Introduction

Vascular endothelial dysfunction plays a critical role in the pathogenesis of atherosclerosis. A wealth of evidence indicates that the endothelium is important in maintaining vascular homeostasis by regulating vasoreactivity, platelet activation, leukocyte adhesion, and smooth muscle cell proliferation and migration (Ryoo et al., 2006). Endothelium-derived NO (nitric oxide) is produced from its precursor, L-arginine, by endothelial NO synthase 3 (eNOS), which has a critical role in the regulation of vascular tone and maintenance of vascular integrity (Ryoo et al., 2008). Arginase and eNOS share the same substrate, L-arginine, and arginase hydrolyzes L-arginine to ornithine and urea as part of the urea cycle. Thus, the balance of arginase and eNOS activities regulates in part vascular endothelial NO production. A high expression of arginase may shift the arginase–NOS balance toward decreased NO production, which is associated with endothelial dysfunction in a number of pathophysiological processes such as aging, diabetes, hypertension and atherosclerosis (Kim et al., 2009).

Previous studies have demonstrated that arginase II is associated with atherosclerosis. Arginase II modulates eNOS activity by regulating intracellular L-arginine bioavailability, which results in atherosclerosis (Ryoo et al., 2006). Recent studies reported that oxidized low-density lipoprotein (oxLDL) decreases endothelial NO in human aortic endothelial cells by increasing arginase II activity reciprocally, and the supply of L-arginine to eNOS is impaired in the presence of oxLDL (Kim et al., 2009). Small interfering RNA against arginase II prevented the oxLDL-mediated increase in arginase II activity and maintained NO production (Kim et al., 2009). Because arginase II is an important regulator of NO production in endothelial cells of humans and other species, suppression of arginase II expression has been effective in preventing endothelial dysfunction and the progression of atherosclerosis (Brandes, 2006). However, the specific mechanisms of the transcriptional regulation of arginase II are still poorly understood.

Poly- (ADP-ribose) transferase/polymerase-1 (PARP1) is a 116-kDa nuclear protein widely known for its DNA-binding properties and its unique enzymatic activity for ADP-ribosylation (Oumouna-Benachour et al., 2007). PARP1 binds cooperatively to different DNA structures with high affinity and takes part in many biological functions, such as DNA repair, replication and recombination, apoptosis and oncogenesis (Hans et al., 2009). Emerging evidence shows PARP1 associated with maintenance of eNOS activity and dyslipidemia-induced endothelial dysfunction (Pacher et al., 2004; Pacher and Szabo, 2007). The possible role of PARP1 in the transcription of specific genes has been investigated by various experimental approaches. PARP1 was identified as a constituent of the positive cofactor-1 complex, which is essential for the activity of several transcription factors (Butler and Ordahl, 1999).

We aimed to determine whether PARP1 is a key factor responsible for the basal transcription of arginase II and for oxLDL-induced upregulation of arginase II. We investigated the core promoter region of arginase II to identify the direct binding transcriptional factors responsible for arginase II basal transcription, as well as oxLDL-mediated increase in arginase II production. We validated these primary findings in both *in vitro* and *in vivo*.

## Materials and Methods

### Plasmid Construction

The promoter region 900 bp upstream of arginase II (from -909 to +5 bp) with serial deletions was inserted into the pGL3-basic vector (Cat#: E1751, Promega, Madison, WI, United States). Specifically, the following regions of the arginase II promoter were constructed: -909, -774, -738, -686, -624, -490, -344, and -175 bp between the multiple cloning sites *XhoI* and *KpnI*. Primers with the restriction sites were used for cloning (**Table 2**). The human PARP1 cDNA was amplified by RT-PCR from RNA of 293T cells. The PCR products were cloned into the pCDNA3.1 (-) expression vector.

### Cell Culture, Transfection and Treatment

All plasmids and the siRNA duplex were transfected with use of Lipofectamine 2000 (Invitrogen, Carlsbad, CA, United States) into human aortic endothelial cells (HAECs). The following siRNA sequences for PARP1 were sense, 5′-rArArUrCrArUrArCrUrCrCrArArGrGrArArGr-CrArUrUrU-3′; and antisense, 5′-rArArArUrGrCrUrUrCrCrUrUrGrGrArGrUrArUrGrArUrUr-3′. To examine the dose-dependent effects, HAECs were treated with 0, 5, 10, 15, 30, and 50 μg/mL oxLDL 24 h before cells were harvested for measurement of target gene mRNA and protein levels. To examine the time-dependent effects, cells were treated for 0, 0.5, 1, 6, 12, and 24 h with 50 μg/ml oxLDL. The following reagents were used for experiments: BEC (10 μm/L), an inhibitor of arginase II; C9873 (10 μm/L), an inhibitor of histone acetytransferase (10 μm/L); PD98059 (20 μm/L), an inhibitor of MEK1/2 phosphorylation; U0126 (10 μm/L), an inhibitor of ERK1/2 phosphorylation; and DPQ (10 μm/L), an inhibitor of PARP-1 (Sigma–Aldrich, St. Louis, MO, United States).

### Luciferase Assay

HEK293 cells (2 × 10^5^ cells) were seeded in 24-well plates, and 24 h later, 0.2 μg of recombinant pGL3 basic vector containing various lengths of the promoter region of ARG2 sequence and 25 ng of pRL-SV40 renilla luciferase vector (Promega) were cotransfected into cells using 4 μg Lipofectamine. After 6 h of incubation, the medium was replaced with the regular medium. After 24 h of transfection, luciferase activity was detected with use of a luminometer (TD-20/20 Turner Designs, Promega) with luciferase assay reagent (Promega) as previously described (Zhang et al., 2008b).

### Purification and Identification of the -774 to -738 bp DNA Element Binding Proteins

The proteins binding to the -774 to -738 bp DNA element were purified by use of a DNA-binding protein purification kit (Roche, Indianapolis, IN, United States) following the manufacturer’s instructions. In brief, the double-stranded oligonucleotide was biotin-labeled for DNA fragments containing the -774 to -738 bp DNA element CTTACAGCTCTTTAGCTTAGAAATAGTCTAAATACG. The binding proteins were digested with trypsin and desalted by use of C18 Ziptip (Millipore, Inc.) and analyzed by 4800 MALDI-TOF-TOF mass spectrometry (MS/MS) (ABI, Foster City, CA, United States) with 0.15 and 0.25 Da mass tolerance for PMF and MS/MS, respectively (Zhang et al., 2009). Identification of binding proteins involved a Group-based Prediction System, and a confidence interval of greater than 99.99% was accepted.

### Docking and Predication of the Binding Sites in -774 to -738 bp DNA Element for PARP1

The coordinate files of the different domains in PARP1 were obtained from the RCSB Protein Data Bank^1^. The PDB entry codes were as follows: 2CR9, the solution NMR structures for the WGR domain; 2DMJ and 2CS2, the solution NMR structures for the first and second Zn-finger domain; and 2RIQ, the x-ray crystal structures of the third Zn-finger domain. The canonical B-DNA 3D structure of the -774 to -738 bp DNA element was constructed with use of 3D-DART as described (Zhang et al., 2008b). The docking of PARP1 and the -774 to -738 bp DNA element involved use of HADDOCK as described (van Dijk et al., 2006).

### ChIP Assay

We used the chromatin immunoprecipitation (ChIP) assay kit (Millipore) as described (Zhang et al., 2008a). Immunoprecipitated DNA and input DNA were quantified with use of the quantitative RT-PCR detection system (Bio-Rad). The relative levels of DNA were normalized to the input DNA and expressed as percentage of the non-treatment control. The PCR products were separated on a 1.5% agarose gel. The following primers were used for the PARP1 binding site in the 5′-flanking region of the human arginase II gene: forward, 5′-GCTTAGAAATAGTCTAAATACG-3′, and reverse, 5′-GCATCAGTGAAATTTGGATTC-3′.

### Quantitative Real-Time RT-PCR

Total RNA from treated cells was extracted with Trizol (Invitrogen) according to the manufacturer’s protocol. The mRNAs were reverse-transcribed with use of the iScript cDNA synthesis kit (Bio-Rad). qRT-PCR involved the iCycler iQ RT-PCR detection system (Bio-Rad). Primers were designed with use of Beacon Designer 2.0 and chemically synthesized (Shanghai BioSune Biotechnology, China). The primer sequences for PARP1 were sense, 5′-CATGACCCTGAGACTGGAAA-3′, and antisense, 5′-GCCAGGCACTCTTAGATACT-3′; arginase II, sense, 5′-GTTTGGGCTGCCACCTAAAAG-3′, and antisense, 5′-CACTAATGGTACCGATTGCC-3′; and the housekeeping gene human β-actin, sense, 5′-CTGGAACGGTGAAGGTGACA-3′, and antisense, 5′-GGGACTTCCTGTAACAATGCA-3′. The mRNA levels were acquired by normalizing the threshold cycle (Ct) of PARP1 to that of β-actin.

### Western-Blot Analysis

Protein extracted from the cultured cells or aortas tissues were separated by 10% SDS-PAGE and transferred to nitrocellulose membranes. The membrane was blocked in 5% non-fat milk in TBS-T buffer and incubated with primary antibodies for PARP1 (Sigma–Aldrich, Cat#: HPA045168), arginase II (Abcam, Cambridge, MA, United States), eNOS (Sigma–Aldrich). The membrane was washed extensively with TBS-T before incubation with secondary anti-rabbit horseradish peroxidase-conjugated antibody. After extensive washing with TBS-T, the membrane was visualized with use of ECL-plus reagents for chemiluminescence detection (Amersham Biosciences).

### Co-immunoprecipitation

To study the interaction between PARP1 and phosphorylated ERK1/2, HAECs were harvested in lysis buffer for immunoprecipitation with a mouse anti-PARP1 antibody (Sigma–Aldrich, Cat#: HPA045168) at 4°C overnight. Prewashed protein A/G beads (100 μl at 50%; Santa Cruz Biotechnology) were then added to the mixture and incubated for 4 h. Beads were washed five times with PBS. Isolated protein complexes were denatured for 5 min at 95°C, analyzed by 7.5% SDS-PAGE, and transferred to PVDF membranes, followed by immunoblotting with anti- phosphorylated ERK1/2 antibodies (Santa Cruz Biotechnology) with detection by enhanced chemiluminescence (Pierce).

### Animal Models

PARP1-/- mice (in a C57BL/6J background) were obtained from Dr. Chang-sun Shao (Rutgers University, Piscataway, NJ, United States) and the ApoE-/- mice were obtained from Jackson Laboratory (Bar Harbor, ME, United States) and kept on a regular diet. Mice were housed in temperature-controlled cages (20–22°C), fed ad libitum, and maintained on a 12 h light/12 h dark cycle. At age 8 weeks, WT, ApoE-/- mice and ApoE-/-PARP-1-/- male mice (*n* = 15) were fed a normal diet (ND) or a high-cholesterol diet (1.25% cholesterol) for 12 weeks. ApoE-/-PARP-1-/- mice were generated by crossing the offspring of ApoE+/-PARP-1+/- mice that resulted from mating their respective homozygous knockouts and were genotyped by PCR. All studies were performed with the approval of the Animal Care and Use Committee of Shandong University. The protocol was approved by this committee.

### Serum Lipid Levels

At the time of tissue harvesting, 0.5–1.0 mL of blood was drawn from the right ventricle of mice with use of heparinized syringes and immediately centrifuged at 4°C. Serum levels of total cholesterol (TC), triglycerides (TGs), low-density lipoprotein cholesterol (LDL-C) and high-density lipoprotein cholesterol (HDL-C) were measured by standard enzymatic methods and commercial kits (Roche Diagnostics, Indianapolis, IN, United States).

### Vasorelaxation Measurement

By use of previously described methods (Winters et al., 2000), the mice were sacrificed, and excised aortas were cut into rings 2–3 mm long; each ring was connected to an isometric force transducer (Multi-Myograph 610M, Danish Myo Technology A/S, Aarhus, Denmark), suspended in an organ chamber filled with 5 ml Krebs-Ringer bicarbonate solution (37°C, pH 7.4; 118.3 mM NaCl, 4.7 mM KCl, 1.2 mM MgSO_4_, 1.2 mM KH_2_PO_4_, 2.5 mM CaCl_2_, 25 mM NaHCO_3_, 0.026 mM EDTA, and 11 mM glucose), and bubbled with 95% O_2_ and 5% CO_2_. Dose responses to vasoconstrictors norepinephrine (NE; 10^-9^–10^-5^ M). Endothelium-dependent vasorelaxant responses to increased doses of acetylcholine (0.1 nM to 100 μM) (Sigma–Aldrich) were measured and expressed as a percentage of post-acetylcholine tension to the post-norepinephrine tension.

### Measurement of NO Production

Nitric oxide production was estimated by flow cytometry with 4,5-diaminofluorescein diacetate used as a reagent to quantify NO. HAECs were transfected with siRNA for PARP1 labeled with FAM for 24 h and then incubated with or without oxLDL for 1 h. After oxLDL treatment, cells were washed twice with phosphate buffered saline (PBS; pH 7.2∼7.4) and then incubated for 40 min with 10 μmol/L 4,5-diaminofluorescein diacetate at 37°C. Cells were washed again with PBS buffer and incubated for an additional 30 min to allow for complete de-esterification of the intracellular diacetates. Fluorescence emission was measured at 515 nm by flow cytometry (FACSCalibur, BD, Sparks, MD, United States). Data were analyzed with use of Cell Quest Pro software (BD, Sparks, MD, United States). NO production in aortic rings was measured by DAF-FM fluorescence as previously described (Hans et al., 2009).

### Immunocytochemistry and Immunohistochemistry of HAECs

Human aortic endothelial cells were fixed in 4% paraformaldehyde and permeabilized in PBS containing 0.5% Triton X-100. Indirect immunofluorescence involved use of monoclonal antibodies against CD31 (Sigma–Aldrich), arginase II (Abcam) and eNOS (Sigma–Aldrich). Goat anti-mouse cyanine Cy2 (green) and donkey anti-rabbit Cy5 (red) secondary antibodies (Jackson Laboratory, Bar Harbor, ME, United States) were used to detect the location and expression of CD31, arginase II and eNOS in HAECs. Nuclei were visualized with 4-6-diamidino-2-phenyl indole-2HCl (DAPI, Santa Cruz Biotechnology, Santa Cruz, CA, United States). Serial sections (6 μm) of mouse aortic arches were fixed in paraformaldehyde (4°C, 15 min) and permeabilized with 0.1% Triton X-100 for 5 min. Sections were incubated with primary antibodies overnight at 4°C, then with a fluorescence dye–conjugated secondary antibody. Laser-scanning confocal microscopy (LSM710, Carl Zeiss, Jena, Germany) involved krypton-argon and helium-neon lasers.

### Statistical Analysis

Data are expressed as mean ± SEM for quantitative measurements. Independent Student’s *t-*test was used for comparison between groups and one-way ANOVA for comparison among multiple groups with post-hoc Bonferroni correction. SPSS v12.0 for Windows (SPSS, Chicago, IL, United States) was used for analysis. *P* < 0.05 was considered significant.

## Results

### PARP1 Directly Binds to the Region -774 to -738 bp at the Arginase II Promoter

**FIGURE 1:**
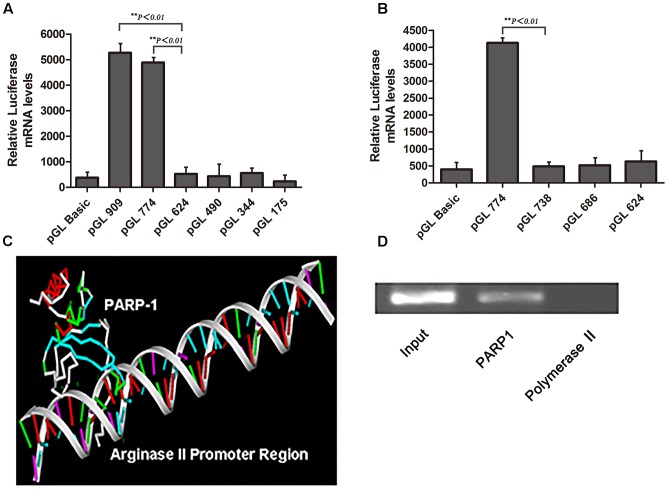
Identification of the core element of arginase II promoter and PARP1 as the binding protein. **(A)** Relative luciferase mRNA expression in human aortic endothelial cells (HAECs) transfected with pGL3-promoter constructs containing DNA fragments serially deleted 900 bp upstream of the arginase II promoter. HAECs transfected with the pGL3-basic vector were the negative control. Data were normalized to the activity of the co-transfected pSV galactosidase plasmid. **(B)** Because the arginase II core promoter was identified in the –774 to –624 bp region, we performed serial deletion of the region with pGL3 basic vectors to construct pGL774, pGL738, pGL686, and pGL624; the basic vector was a control. The luciferase mRNA levels were normalized to that of the co-transected pSV galactosidase plasmid. **(C)** PARP1 binding to the –774 to –738 bp region by mass spectrometry through DNA-affinity purification. 3D-DART and HADDOCK software were used to analyze the spatial structure of PARP1 and its promoter region, and the PARP1 binding site was identified as “ACAGCT” in the –774 to –738 bp fragment. **(D)** ChIP assay of PARP1 activity, with polymerase II as a negative control. The input control represents the whole nuclear proteins of HAECs. PCR analysis involved primers that covered the –774 to –738 bp region of the arginase II promoter after ChIP reactions.

To clarify the core regulatory element in the promoter region of arginase II, HAECs were transfected with several pGL3 reporter constructs containing progressively deleted 5′-flanking regions from -909 to +5 bp of the arginase II promoter region, then luciferase mRNA levels were measured. Relative luciferase mRNA level was reduced by 90% (*p <* 0.01) with pGL-624 transfection as compared with pGL-909 or pGL-774 transfection, so the core promoter element was located at -774 to -624 bp (**Figure 1A**). Then with pGL3 reporter constructs containing a series of deletions from -744 to -624 bp of the promoter region, luciferase mRNA level was decreased by 95% (*p <* 0.01) with pGL-738 transfection as compared with pGL-774 transfection (**Figure 1B**). Therefore, the 40-bp core active element, located at -774 to -738 bp of the arginase II promoter region, plays a critical role in the arginase II transcription process.

Nuclear proteins binding with a biotin-labeled probe, covering the core elements of -774 to -738 bp, were separated by SDS-PAGE and identified by MALDI-TOF-TOF MS/MS. PARP1 was chosen from several identified proteins on the basis of the spectra of 3 peptides with the highest MASCOT score, 159.

Analysis of the spatial structure of the PARP1 binding domain in the -774 to -738 bp fragment by use of HADDOCK software suggested that PARP1 might bind with the ACAGCT site (-771 to -766 bp) in the -774 to -738 bp fragment of the arginase II promoter (**Figure 1C**). ChIP assay was used to confirm the binding of PARP1 with the arginase II promoter region. PCR analysis with specific primers covering -774 to -738 bp of the arginase II promoter region revealed an amplified band in the PARP1 group with anti-PARP1 antibody. In contrast, no amplification band was observed in the -774 to -738bp region in the control group with polymerase II antibody (**Figure 1D**). Therefore, PARP1 specifically binds to the -774 to -738 bp region of the arginase II promoter.

### PARP1 Regulates the Basal Transcription of Arginase II in HAECs

**FIGURE 2:**
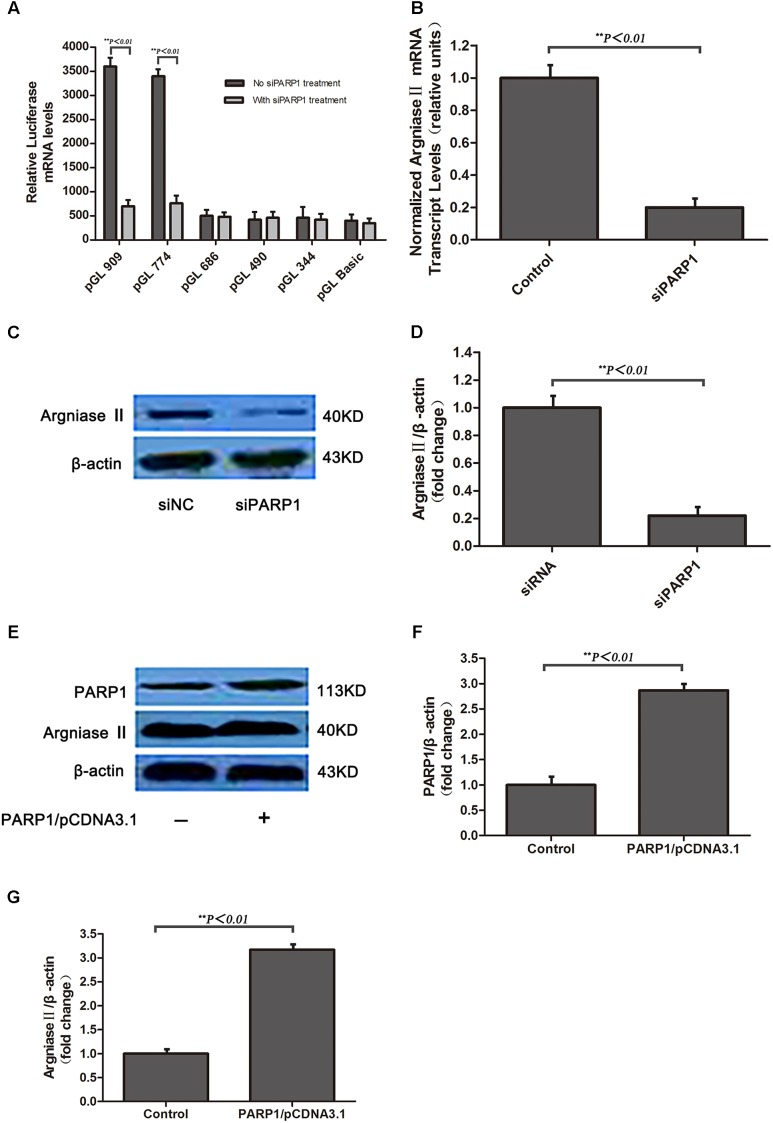
Arginse II expression after PARP1 silencing and overexpression in human aortic endothelial cells (HAECs). HAECs were treated with PARP1 siRNA or PARP1/CDNA3.1. **(A)** Serially deleted pGL3 vectors were transfected into HAECs, then luciferase mRNA level was assessed after transfection of PARP1 siRNA (siPARP1). **(B)** Quantitative real-time RT-PCR analysis of mRNA level of arginase II with or without siPARP1. **(C)** Western blot analysis of protein levels of PARP1 and arginase II with siPARP1 or negative control (siNC) and **(D)** quantification of PARP1 expression. β-actin was the internal control. **(E)** Western blot analysis of protein levels of arginase II and PARP1 with PARP1/pCDNA3.1 or pCDNA3.1 vector transfection and **(F)** quantification of PARP1 expression. Treatment with pCDNA3.1 vector was the control. **(G)** Quantitative analysis of arginase II expression in **E**. Data are mean ± SEM, *n* = 3 per group.

To investigate whether PARP1 is the transcription factor of arginase II, pGL3-basic vectors containing several promoter regions of arginase II were transfected into HAECs with or without PARP1-specific siRNA treatment. Luciferase mRNA level was suppressed after pGL-909 and pGL-774 transfection by nearly 80% with PARP1 siRNA treatment (*p <* 0.01) but did not differ with or without PARP1 siRNA treatment after pGL-686, pGL-490, and pGL-344 transfection (**Figure 2A**).

RT-PCR and western blot analysis revealed arginase II mRNA and protein expression lower, by 80 and 75%, respectively, with PARP1 siRNA treatment than control treatment (**Figures 2B–D**). PARP1 overexpression induced by transfection of the PARP1/pCDNA3.1(-) vector into HAECs markedly increased PARP1 and arginase II expression (*p <* 0.01, **Figures 2E–G**) as compared with the control group. Thus, PARP1 as a transcription factor binds the arginase II promoter and is responsible for the basal transcription of arginase II.

### OxLDL Upregulates PARP1 Expression *in vitro* and *in vivo*

**FIGURE 3:**
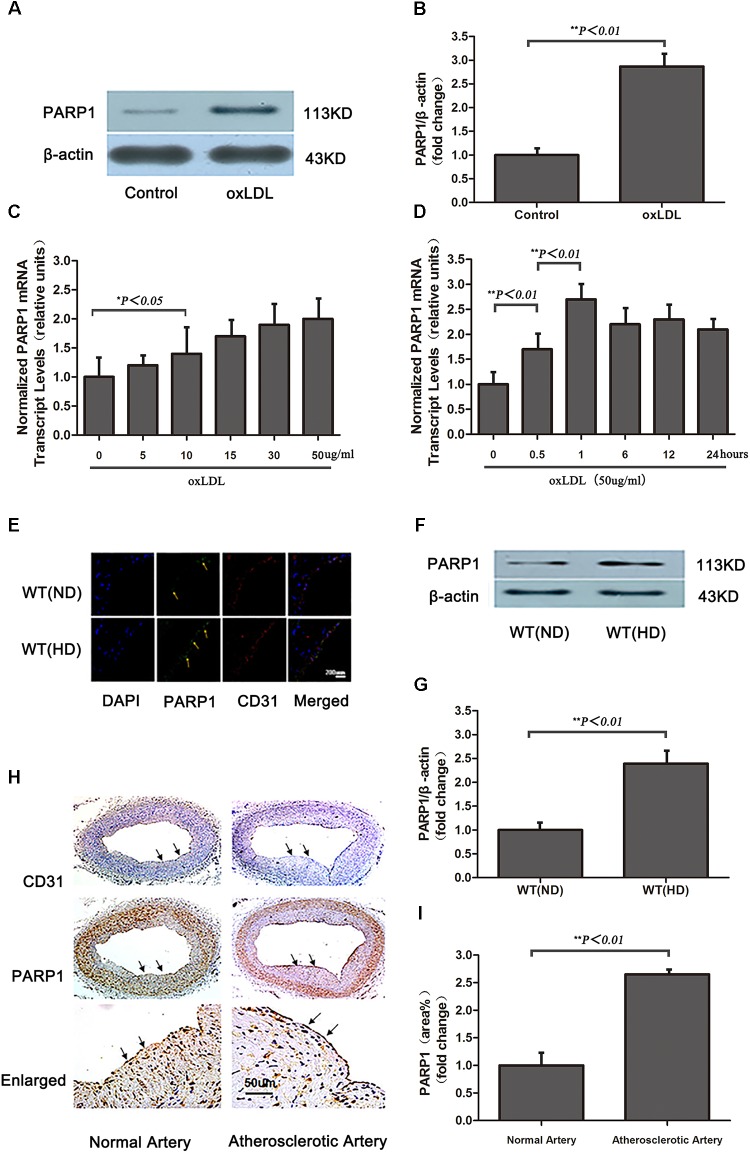
Oxidized low-density lipoprotein upregualates PARP1 expression *in vitro* and *in vivo.* **(A)** Western blot analysis of PARP1 protein expression in HAECs after oxLDL stimulation for 24 h and **(B)** quantification. **(C)** Quantitative real-time RT-PCR analysis of dose-dependent effects of oxLDL on mRNA level of PARP1 and **(D)** time-dependent effects. Data are mean ± SEM, *n* = 3 per group. **(E)** Immunohistochemistry of PARP1 protein expression in endothelium of artery cross sections of wild-type (WT) aortas with a normal diet (ND) or high-cholesterol diet (HD) for 12 weeks. Green fluorescence represents PARP1 activity (arrows show endothelium of artery), and blue DAPI-stained nuclei (*n* = 6 for each group). **(F)** Western blot analysis of PARP1 protein expression in arteries from WT mice fed a ND or HD and **(G)** quantification. **(H)** Immunohistochemistry of PARP1 protein expression in endothelium of artery cross sections in human normal or atherosclerotic arteries. Upper panel is endothelium (arrows show endothelium of artery) with CD31 antibody staining; middle panel is PARP1 protein level in endothelium (arrows show endothelium of artery) by PARP1 antibody staining; and lower panel is enlarged and **(I)** quantification (*n* = 3 for each group).

To evaluate whether oxLDL stimulates PARP1 production, HAECs were treated with 50 μg/ml oxLDL for 24 h and showed markedly increase in PARP1 protein expression as compared with control treatment (*p <* 0.01, **Figures 3A,B**). The increased expression of PARP1 mRNA in HAECs began as early as 0.5 h and lasted for 24 h (**Figures 3C,D**). The maximal PARP1 mRNA level was observed at 1 hr after oxLDL stimulation and was about 2.5-fold of that in the control group (*p* < 0.01, **Figure 3C**). PARP1 mRNA levels were increased dose-dependently 1 h after oxLDL treatment, with maximal effect at 50 μg/mL oxLDL with more than 2.5-fold increase versus the control group (*p <* 0.01, **Figure 3D**). Therefore, we used 50 μg/ml oxLDL for 1 h to induce PARP1 expression level.

Because a high cholesterol diet may lead to increased serum oxLDL level *in vivo*, PARP1 expression was measured in aortas of WT mice fed a high cholesterol diet (1.25% cholesterol) for 12 weeks (serum lipid levels in **Table 1**). Confocal microscopy and western blot revealed that PARP1 expression markedly increase in the endothelium of aortas with a high-cholesterol than ND (*p <* 0.01, **Figures 3E–G**). Similar results were observed in human normal and atherosclerotic arteries (*p <* 0.01, **Figures 3H,I**). Thus, oxLDL may stimulate PARP1 production *in vitro* and *in vivo*.

**Table 1:** Serum Lipid levels in 4 groups of mice after 12 weeks’ feeding.

	WT ND (*n* = 10)	WT HC (*n* = 10)	PARP1-/- ND (*n* = 8)	PARP1-/- HC (*n* = 8)
TC (mmol/L)	1.53 ± 0.24	2.60 ± 0.19	2.10 ± 0.31	3.63 ± 0.28^##^
HDL (mmol/L)	1.18 ± 0.14	2.34 ± 0.15	1.82 ± 0.17^∗∗^	3.48 ± 0.22^∗∗##^
LDL (mmol/L)	0.51 ± 0.08	0.98 ± 0.11^∗∗^	0.61 ± 0.11	1.86 ± 0.12^∗∗##^
TG (mmol/L)	1.16 ± 0.24	0.69 ± 0.12^∗∗^	0.76 ± 0.13^∗∗^	0.75 ± 0.14^∗∗^

*Data are mean ± SEM. ^∗∗^*P* < 0.001 vs. wild-type (WT) ND group. ^##^*P* < 0.01 vs. WT high cholesterol diet (HC) group. HC, high cholesterol diet; PARP, poly-(ADP-ribose) transferase/polymerase-1; TC, total cholesterol; HDL, high-density lipoprotein; LDL-low-density lipoprotein; TG, triglicerides.*

**Table 2:** Primers with restriction site (underlined) for constructing pGL3-arginase II promoter region vectors.

Forward	
pGL909	5′-CTCGAGGGGAAAATTACTTATGTC-3′
pGL774	5′-CTCGAGCTTACAGCTCTTTAGCTTAGAAATAGTC-3′
pGL738	5′-CTCGAGTTGCTATATTTGCTTTTT-3′
pGL686	5′-CTCGAGTTAAAAACATCTAAAAGG-3′
pGL624	5′-CTCGAGGGTAGATGGCTCTTTATAAACGTG-3′
pGL490	5′-CTCGAGGGATCCTGGCTTTGACTAGGCAGG-3′
pGL344	5′-CTCGAGCGAAGGGACACACCTGTTGGTCAC-3′
pGL175	5′-CTCGAGGAAGGTGTGCCGGGGGCTGGTTGGA-3′
Reverse	5′-GGTACCGACATGATCCGCAGCACTGAGAACT-3′

### PARP1 Mediates oxLDL-Enhanced Arginase II Expression

**FIGURE 4:**
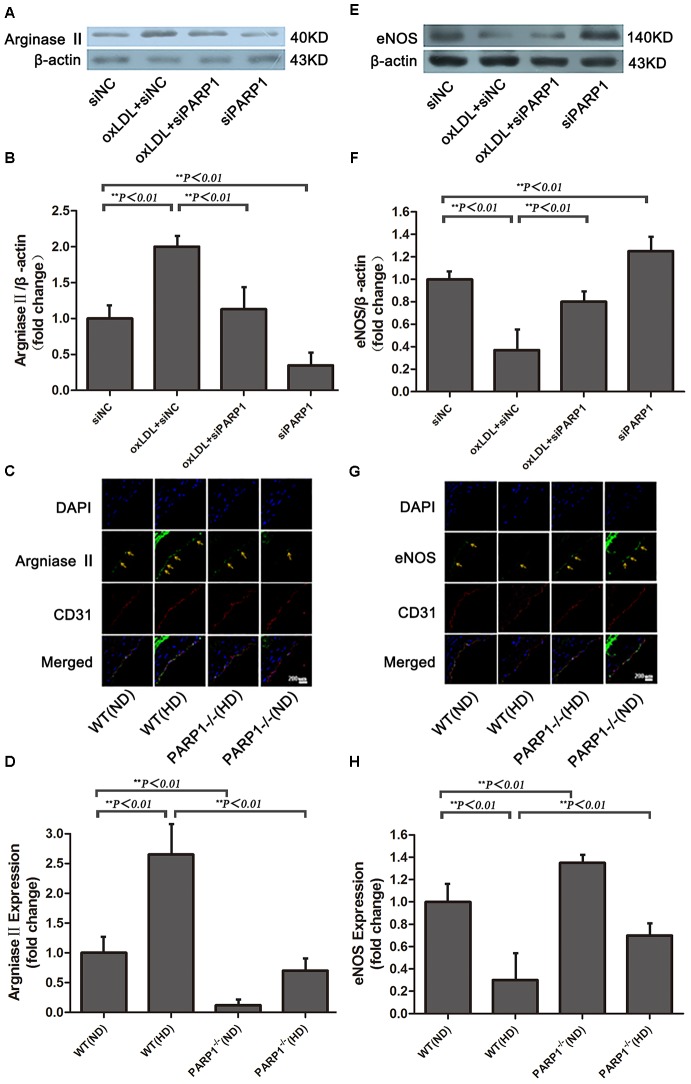
Arginase II and eNOS expression after PARP1 inhibition. **(A)** Western blot analysis of arginase II protein expression in HAECs and **(B)** quantification. **(C)** Immunofluorescence double staining of arginase II (green) or CD31 (red) in arteries of WT and PARP1–/– mice fed an ND or HD for 12 weeks and **(D)** quantification (*n* = 6 for each group, arrows show endothelium of artery). Nuclei were stained by Hoechst (blue), and yellow indicates merged images. **(E)** Western blot analysis of eNOS expression in HAECs and **(F)** quantification. **(G)** Immunofluorescence double staining of eNOS (green) or CD31 (red) in arteries of WT and PARP1–/– mice fed a ND or HD and **(H)** quantification. Nuclei were stained by Hoechst (blue), and yellow indicates merged images. (*n* = 6 for each group, arrows show endothelium of artery).

At 24 h after transfection of PARP1 specific siRNA into HAECs, cells were treated with oxLDL for 1 h. oxLDL stimulation induced more than two-fold increase in arginase II protein expression as compared with control treatment (*p <* 0.01) (**Figures 4A,B**). However, PARP1 siRNA treatment reversed most of the oxLDL-mediated increase in arginase II expression as compared with oxLDL treatment alone (*p <* 0.01). In contrast, arginase II level did not differ between oxLDL plus PARP1 siRNA and control treatment. PARP1 silencing alone decreased arginase II expression to about 25% of that with control treatment (*p <* 0.01).

PARP1-/- and wild-type (WT) mice were used to investigate the role of PARP1 in regulating arginase II expression. Arginase II expression in WT aortas was higher with a high-cholesterol than ND (*p <* 0.01) (**Figures 4C,D**), which is consistent with our *in vitro* results. In contrast, arginase II expression was lower in PARP1-/- than WT aortas with the same high cholesterol diet (*p <* 0.01). Furthermore, arginase II expression was significantly lower in PARP1-/- than WT aortas with the same ND (*p <* 0.01). However, arginase II expression did not differ between PARP1-/- aortas with a high cholesterol diet and WT aortas with a ND. Thus, PARP1 as a transcription factor mediated oxLDL enhanced arginase II expression both *in vitro* and *in vivo.*

### PARP1 Silencing Restores eNOS Expression Downregulated by oxLDL Stimulation

After oxLDL stimulation, eNOS expression in HAECs was downregulated by more than 60% as compared with control treatment (*p <* 0.01) (**Figures 4E,F**). However, the suppressive effect of oxLDL on eNOS expression was largely reversed by PARP1 siRNA treatment (*p <* 0.01). Furthermore, PARP1 silencing alone significantly upregulated eNOS protein expression as compared with control treatment (*p <* 0.01).

To study whether PARP1 deficiency may increase eNOS expression *in vivo*, we used confocal microscopy to determine the level of eNOS expression in PARP1-/- and WT aortas with a normal or high-cholesterol diet. eNOS expression in WT aortas was lower with a high-cholesterol than ND (*p <* 0.01) (**Figures 4G,H**) but was significantly higher in PARP1-/- than WT aortas with the same high-cholesterol diet (*p <* 0.01). Similarly, eNOS expression level was higher in PARP1-/- than WT aortas with the same ND (*p <* 0.01). These data support the notion that PARP1 mediates the suppressive effects of oxLDL on eNOS expression by regulating arginase II production.

### PARP1-/- Mice Show Improved Vascular Endothelial Function and Suppressed Atherogenesis

**FIGURE 5:**
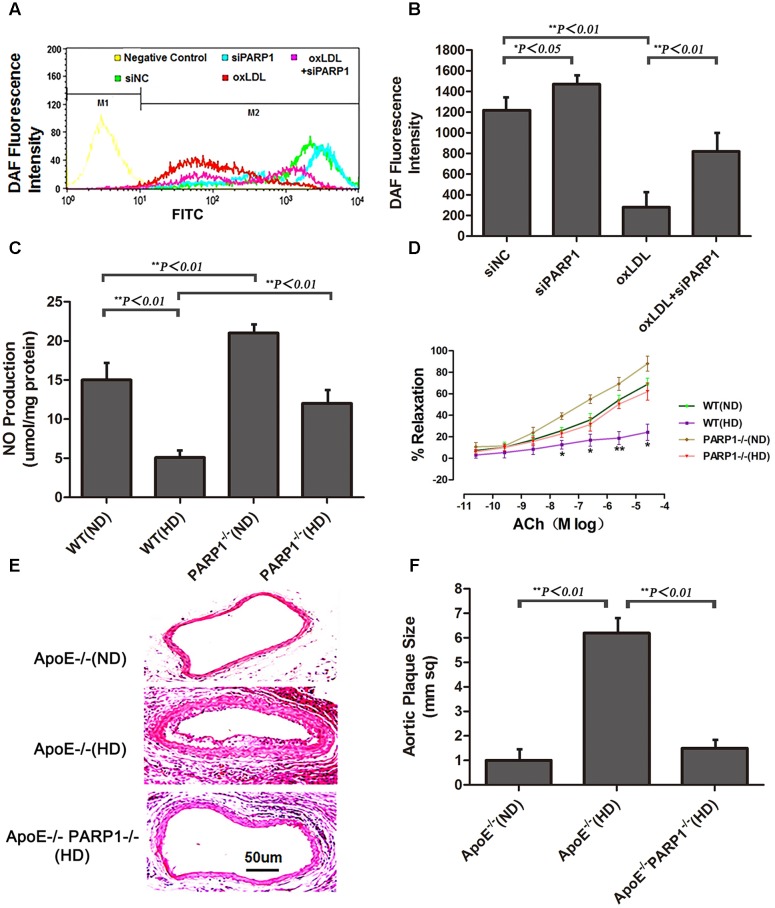
Endothelial function and atherogenesis after PARP1 deletion. **(A)** Flow cytometry of NO expression in HAECs with or without siPARP1 after oxLDL stimulation (50 μg/ml for 1 h), stained with the NO-sensitive fluorescent dye DAF, and **(B)** quantification. Data are mean ± SEM, *n* = 3 per group. **(C)** Vascular NO was measured in WT and PARP1-/- aortas with an ND or HD for 12 weeks (*n* = 6 for each group). **(D)** Endothelium-dependent vasorelaxation of aortas isolated from WT and PARP1-/- mice after 12 weeks of ND or HD feeding. Association of increased doses of acetylcholine (Ach) and vasorelaxation after administration of norepinephrine (10^-5^ mol/L). Data are mean ± SEM; ^∗^*P <* 0.05 for WT aortas with an HD vs. all other groups. (*n* = 6 for each group). **(E)** Hematoxylin and eosin staining of carotid arteries from ApoE-/- and ApoE-/- PARP1-/- mice fed a ND or HD for 12 weeks. **(F)** quantification of aortic plaque size.

Compared with control HAECs, PARP1-silenced HAECs showed substantially increased NO production, as determined by use of the NO sensitive fluorescent probe DAF-FM (*p <* 0.05) (**Figures 5A,B**). With oxLDL stimulation, PARP1 siRNA treatment reversed most of the suppressive effects of oxLDL on NO production (*p <* 0.01). NO production was significantly higher in WT aortas with a normal than high-cholesterol diet (*p <* 0.01) and higher in PARP1-/- than WT aortas with the same high-cholesterol diet (*p <* 0.01) or the same ND (*p <* 0.01) (**Figure 5C**). Isometric tension measurement showed markedly enhanced vasorelaxation in aortic rings from PARP1-/- as compared with WT aortas with the same ND (*p <* 0.05) (**Figure 5D**). Vasorelaxation of the aortic rings was significantly attenuated in WT aortas with a high-cholesterol as compared with ND (*p <* 0.05). However, aortic rings showed substantially greater vasorelaxation in PARP1-/- than WT aortas with the same high-cholesterol diet (*p <* 0.05). To confirm the role of PARP1 in progression of atherosclerotic plaques, we generated homozygous PARP1/ApoE double-deletion mice (PARP-1-/-ApoE-/-). ApoE-/- and PARP1-/-ApoE-/- mice were fed a normal or high-cholesterol diet, then carotid arteries were examined. Plaque formation was markedly lower in PARP1-/-ApoE-/- than ApoE-/- aortas with a high-cholesterol diet as compared with ApoE-/- aortas with a ND (**Figures 5E,F**). These results clearly support the hypothesis that PARP1 regulation of arginase II plays an important role in improved vascular endothelial function and atherogenesis.

### OxLDL Upregulates Arginase II Expression by Phosphorylated ERK2 and PARP1 Interaction

**FIGURE 6:**
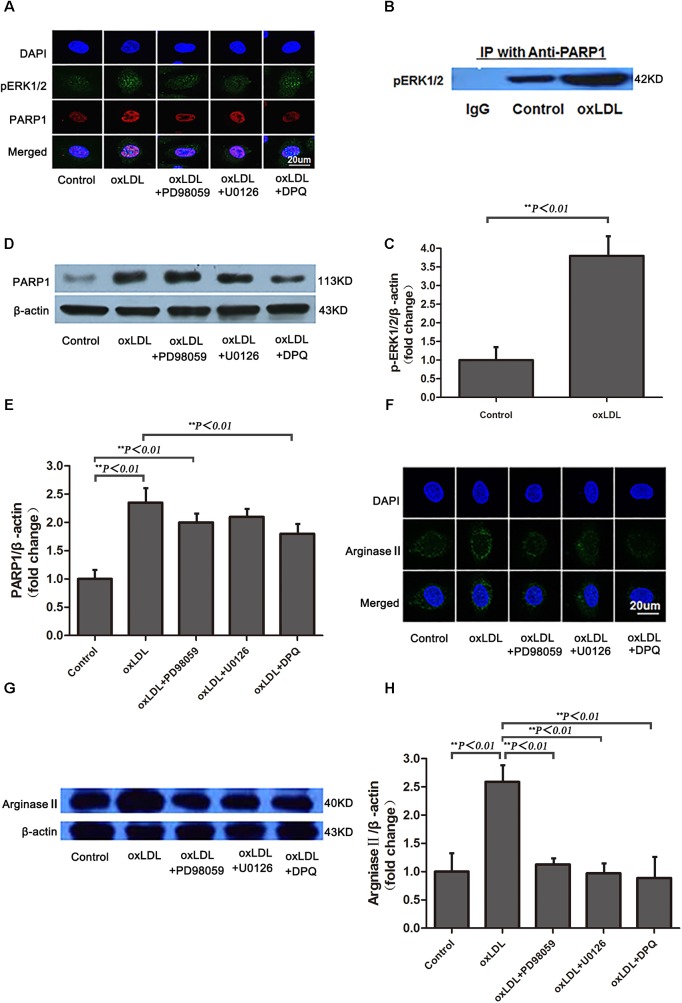
Phosphorylated ERK1/2 expression and nuclear translocation after oxLDL stimulation in HAECs. **(A)** Immunofluorescence staining of pERK1/2 and PARP1 and DAPI (overlay) with or without oxLDL in the presence or absence of MEK (PD98059), ERK1/2 (U0126) and PARP1 (DPQ) inhibitors. **(B)** Western blot analysis of co-immunoprecipitation (IP) of pERK1/2 expression with anti-PARP1 antibodies in HAECs and **(C)** quantification. Data are mean ± SD of 3 independent experiments. **(D)** PARP1 expression and **(E)** quantification; and **(F)** Immunofluorescence staining of arginase II and DAPI (overlay) with or without ox-LDL in the presence or absence of MEK (PD98059), ERK1/2 (U0126) and PARP-1 (DPQ) inhibitors. **(G)** Western blot analysis of arginase II expression and **(H)** quantification. Data are mean ± SEM, *n* = 3 per group.

Previous studies have demonstrated that phosphorylated ERK2 is an activator of PARP-1 with interaction (Cohen-Armon et al., 2007). To investigate the intracellular signaling mechanisms among oxLDL, ERK, PARP1 and arginase II, HAECs were treated with oxLDL with or without PD98059, an inhibitor of MEK1/2 phosphorylation; U0126, an inhibitor of ERK1/2 phosphorylation; or DPQ, an inhibitor of PARP1. ERK1/2 was phosphorylated by upstream kinase MEK1/2, and phosphorylated ERK2 translocated from the cytoplasm to the nucleus after oxLDL stimulation (**Figure 6A**). PD98059 and U0126 but not DPQ inhibited ERK2 translocation in HAECs. Phosphorylated ERK2 was detected in proteins immunoprecipitated by anti-PARP1 antibodies, and a higher level of phosphorylated ERK2 was found with oxLDL than control treatment (*p <* 0.01; **Figures 6B,C**). As well, PARP1 expression was significantly increased by oxLDL treatment but substantially suppressed by PD98059 and U0126 treatment (**Figures 6D,E**). Confocal microscopy and western blot analysis revealed arginase II expression lower with PD98059, U0126 and DPQ than control treatment (**Figures 6F–H**).

### OxLDL Upregulates Arginase II Expression Through PARP1 Mediated Chromatin Remodeling

**FIGURE 7:**
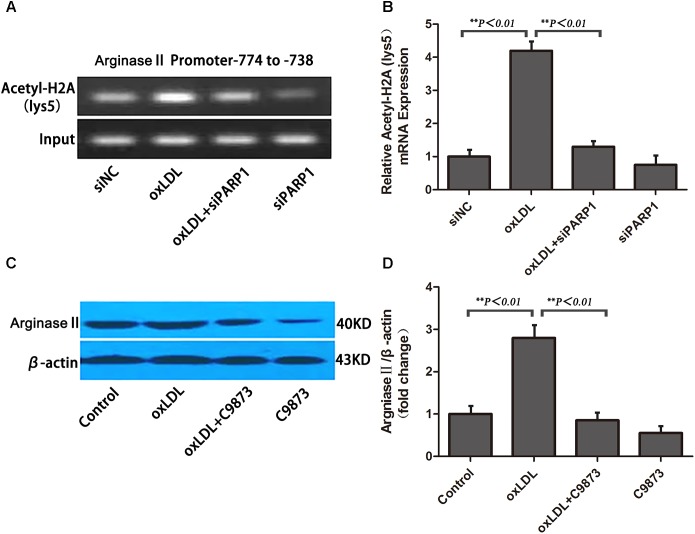
PARP1 Regulates arginase II production by inducing histone acetylation at the arginase II core promoter element in HAECs. **(A)** ChIP assay of acetyl-histone 2A to pull down nucleosomes extracted from HAECs with oxLDL or siPARP1 and **(B)** quantification. Genomic DNA was amplified with primers for the –774– to –738–bp region at the arginase II promoter. **(C)** Western blot analysis of arginase II protein level with C9873, inhibitor of histone acetyltransferase and **(D)** quantification. Data are the mean ± SEM, *n* = 3 per group.

To ascertain the role of PARP1 in histone modification after oxLDL treatment at the core promoter region of arginase II, we used specific primers covering -774 to -738 bp of the arginase II promoter region for PCR reaction. Histone 2A (H2A) lysine 5 within the -744 to -738 bp region was acetylated and enhanced by oxLDL treatment (*p <* 0.01) (**Figures 7A,B**). The acetylation of H2A lysine 5 was deacetylated with PARP1 siRNA. Use of C9873, an inhibitor of histone acetyltransferase, to inhibit the acetylation of H2A lysine 5 suppressed arginase II expression, and the upregulation effects of oxLDL on arginase II were significantly reversed (*p <* 0.01) (**Figures 7C,D**).

## Discussion

In the present study, we identified PARP1 as a transcription factor that directly binds the arginase II promoter element of -774 to -738 bp involved in the regulation of arginase II expression. We further demonstrated that PARP1 was responsible for vascular endothelial dysfunction in mice fed a high-cholesterol diet by upregulating arginase II expression; PARP1 gene silencing largely normalized the vascular endothelial dysfunction in mice. Thus, PARP1 as a transcription factor may regulate arginase II expression and vascular endothelial function in the development of atherosclerosis.

Nitric oxide plays a critical role in preserving vascular homeostasis (Ryoo et al., 2006). Endothelial dysfunction resulting from impaired NO synthesis is a central feature in many vascular disorders (Ryoo et al., 2006). Emerging evidence indicates that endothelial arginase II is a crucial mediator of endothelial dysfunction and a potential therapeutic target for atherosclerosis (Ryoo et al., 2008). Upregulating arginase II inhibits eNOS mediated NO synthesis and may contribute to endothelial dysfunction in hypertension, aging, ischemia and diabetes (Brandes, 2006; Oumouna-Benachour et al., 2007). However, the molecular mechanisms underlying the regulation of arginase II expression are still unknown. In this study, PARP1 interacted both *in vitro* and *in vivo* with the arginase II promoter region of -774 to -738 bp and as a transcription factor contributed to the basal transcriptional regulation of arginase II expression. We found that PARP1 bound with the site of ACAGCT on the arginase II promoter element of -774 to -738 bp, as shown by MS/MS TOF, ChIP assay and use of HADDOCK, and acted as a transcription activator of arginase II. *In vitro* siRNA inhibition of PARP1 suppressed the luciferase activity of the arginase II promoter region and the expression of arginase II protein in HAECs. Moreover, we found low expression of arginase II in PARP1-/- mice fed a high-cholesterol diet. However, overexpression of PARP1 in HAECs markedly enhanced arginase II expression. Our study provides convincing evidence that PARP1, as a transcription factor, mediates arginase II expression and establishes a biological linkage among PARP1, arginase II, eNOS expression and NO production.

A major role for arginase II in the regulation of NO synthesis is to compete with eNOS by limiting the L-arginine substrate, thus leading to decreased NO production. Therefore, arginase II has been implicated in the pathogenesis of a number of NO dysregulation-related diseases (Ryoo et al., 2006). Administration of oxLDL increases the availability and activity of arginase II in atherogenic mice, leads to impaired NO signaling and enhanced reactive oxygen species production as a consequence of eNOS uncoupling, (Ma and Adjei, 2009) and results in endothelial dysfunction and increased vascular stiffness. Thus, clarifying the molecular mechanisms of oxLDL regulation on arginase II expression is important. We revealed a novel mechanism through which PARP1 mediates oxLDL-enhanced arginase II expression and contributes to endothelial dysfunction. *In vitro* oxLDL stimulation enhanced PARP1 expression in a time- and dose-dependent manner in HAECs, which in turn increased the expression of arginase II. Furthermore, PARP1 and arginase II were significantly upregulated in WT mice fed a high-cholesterol diet. More importantly, high levels of arginase II stimulated by oxLDL *in vitro* or a high cholesterol diet *in vivo* were markedly attenuated by PARP1 siRNA treatment in HAECs and by a high cholesterol diet in *PARP1-/-* mice, respectively. These data strongly support that PARP1 plays a key role in oxLDL-mediated upregulation of arginase II.

Recent evidence indicated that PARP1 gene deletion was associated with maintenance of eNOS activity against dyslipidemia-induced endothelial dysfunction (Pacher et al., 2004). However, the detailed mechanisms of this therapeutic effect remain unknown. We found that PARP1 deficiency suppressed arginase II expression, enhanced eNOS expression and improved NO production and endothelial function. To clarify the relationship among PARP1, arginase II, eNOS, NO and endothelial function *in vivo*, we used PARP1-/- mice, an animal model for cancer research (Ma and Adjei, 2009), with or without a high-cholesterol diet. PARP1 gene silencing *in vitro* and PARP1 deficiency *in vivo* were associated with decreased arginase II expression, increased eNOS expression and NO production, and improved endothelial function. Of note, PARP1 gene deficiency normalized the high-cholesterol-diet–induced NO reduction and endothelial dysfunction in mice, possibly by maintaining eNOS activity. These findings clearly demonstrate the unique and powerful effects of PARP1 gene therapy in maintaining normal endothelial function, for a promising therapeutic target for atherosclerosis.

As well, we found that oxLDL induced nuclear migration of phosphorylated ERK2, which directly interacted with PARP1 and greatly enhanced PARP1 interaction and was followed by remodeled histone acetylation to activate arginase II transcription. The PARP1 signal pathway is DNA dependent, and only recently was a DNA-independent PARP1 signal pathway reported, one that interacts with phosphorylated ERK2 (Cohen-Armon et al., 2007). Because oxLDL may activate multiple signaling pathways, including p38, ERK1/2 and p44/42 MAPK (Mehta et al., 2006), our data suggest that phosphorylated ERK2 may be involved in oxLDL-mediated upregulation of PARP1 and arginase II. Previous studies showed that PARP1 is the chromatin-modifying enzyme known to bind the promoter and regulates the gene transcription (Cohen-Armon et al., 2007). Emerging evidence indicated that nuclear PARP1 plays a role in gene transcription associated with components of ATP-dependent chromatin remodeling complexes (Essler et al., 1999; Percipalle and Visa, 2006; Shen et al., 2010; Sun et al., 2011). In our study, mass spectrometry and immunoprecipitation showed that oxLDL enhanced pERK2 interaction with nuclear PARP1 and caused histone acetylation. These biochemical studies suggest that pERK2 and PARP1 physically form a chromatin-remodeling complex on the arginase II promoter to regulate gene transcription. Our data demonstrated acetylation of H2A lysine 5 on the arginase II promoter region of -774 to -738 bp with oxLDL stimulation, and the acetylation process could be deacetylated by the PARP1 inhibitor. In addition, the expression of arginase II was suppressed by the histone acetytransferase inhibitor. oxLDL induced ERK1/2 phosphorylation and migration into nuclei, followed by a direct binding of PARP1 and the arginase II promoter, thus leading to H2A lysine 5 acetylation and activation of the arginase II transcriptional process.

In summary, we demonstrate that PARP1 is a key factor responsible for arginase II basal transcription and oxLDL-mediated upregulation of arginase II. PARP1 deficiency leads to suppressed arginase II expression, enhanced eNOS expression and improved NO production and endothelial function. The underlying mechanisms involve ERK1/2 phosphorylation and migration into nuclei, as well as H2A lysine 5 acetylation. Thus, PARP1 may offer a novel and promising therapeutic target for atherosclerosis.

## Author Contributions

QW and TZ designed and performed the experiments and wrote the manuscript. WZ, WY, BL, and ZW performed the experiments. WQ and QL participated in the discussion during the project design and revised and edited the manuscript. AW and MZ designed the experiments and wrote the manuscript.

## Conflict of Interest Statement

The authors declare that the research was conducted in the absence of any commercial or financial relationships that could be construed as a potential conflict of interest.

## Footnotes

1 http://www.nmr.chem.uu.nl/haddock
